# Editorial: The Olivo-Cerebellar System

**DOI:** 10.3389/fncir.2015.00066

**Published:** 2016-01-12

**Authors:** Egidio D'Angelo, Elisa Galliano, Chris I. De Zeeuw

**Affiliations:** ^1^Neurophysiology Unit, Department of Brain and Behavioral Sciences, University of PaviaPavia, Italy; ^2^Brain Connectivity Center, Neurophysiology, IRCCS C. Mondino Neurological InstitutePavia, Italy; ^3^MRC Centre for Developmental Neurobiology, King's College LondonLondon, UK; ^4^Department of Neuroscience, ErasmusMCRotterdam, Netherlands; ^5^Department of Cerebellar Coordination and Cognition, Netherlands Institute for NeuroscienceAmsterdam, Netherlands

**Keywords:** cerebellum, inferior olive, synaptic plasticity (LTP/LTD), purkinje cell, granular layer, deep cerebellar nucleus

Studies on the olivo-cerebellar system have rapidly advanced over the past decade, leading to new insight in the structural and functional properties of its synapses, neurons, intrinsic circuits, and connectivity with the rest of the brain. As in many other fields of neuroscience, it is becoming more and more appropriate to try to bring our understanding at the level of individual synapses and neurons to that of ensemble activity, circuits, and behavior. This Editorial aims to facilitate this process by ordering the 26 contributions of this special issue of Frontiers in Brain Microcircuits Series from studies on the development and structure of synaptic contacts to those on the function of local microcircuits and network plasticity as well as the olivo-cerebellar system as a whole. More specifically, we highlight here the main points of the chapters on development, circuit organization and structural plasticity of various types of neurons in the olivo-cerebellar system (A); the chapters on their basic activity and synaptic plasticity (B); the chapters on the relevance of the emerging network patterns in the olivo-cerebellar system (C); the chapters on current high-level theories of motor learning (D); and the chapters on the overall role of the olivo-cerebellar system in the integration of sensorimotor control and cognition (E).

## (A) Development, circuit organization, and structural plasticity of the olivo-cerebellar system

The development and architecture of the olivo-cerebellar afferents, the climbing fibers, are described in great detail by Reeber and colleagues and Fujita and Sugihara. Indeed, attempts to relate the climbing fiber branching patterns to the development of cerebellar compartmentalization and lobulation will help us to untangle the organization of the cerebellar cortex at the functional level. Interestingly, the climbing fiber system is not only highly plastic during development, but also following degeneration of Purkinje cells and/or their afferents. Grasselli and Strata highlight how this process depends on the growth-associated protein GAP-43 in olivary neurons, while Mishina and colleagues show how postsynaptic GluRδ2 plays a pivotal role in territory control of the Purkinje cell spines by the parallel fibers versus that of the climbing fibers through trans-synaptic interaction with presynaptic neurexins (NRXNs) and cerebellin 1. The compartmental restriction in sagittal zones, which is evident in the climbing fiber system, apparently also provides a framework for both the excitatory and inhibitory interneurons in the cerebellar cortex in that their axons mostly remain within the same zonal boundaries (Consalez and Hawkes). However, in terms of direct appositions there is a clear distinction between the interneurons in the granular layer, which do not show direct contact with climbing fibers, and those in the molecular layer, which do show adjacent climbing fiber varicosities (Galliano et al.).

## (B) Neuronal activity and synaptic plasticity

The activity at the input stage of cerebellum plays an important role in determining the spatiotemporal patterns of simple spike activity that are ultimately generated by Purkinje cells. Gandolfi and colleagues show how resonance in the granular layer can be sustained at the theta-frequency range by K slow (M-like), KA, and Na-persistent currents and thereby improve spike timing at the millisecond time-scale. In addition, the same lab illustrates how the Golgi cells can fine-tune the spatiotemporal organization of granular layer activity by generating dense center-surround clusters of granule cell activity and implementing combinatorial operations on multiple mossy fiber inputs (D'Angelo et al.), regulating transmission gain and cut-off frequency, controlling spike timing and burst transmission, and determining the intensity and duration of mossy fiber to granule cell plasticity.

Importantly, van Beugen and colleagues were the first to show in awake behaving mammals that the high instantaneous firing frequency of mossy fiber bursts can be reliably transferred to individual granule cells (up to about 800 Hz) and from there via the parallel fibers to Purkinje cells, inducing a heterogeneous short-lived facilitation to ensure signaling within the first few spikes. To what extent the activity in the parallel fibers will be subsequently depressed or potentiated in the Purkinje cells depends on the temporal relation with the climbing fiber activity, implying a non-Hebbian form of spike-timing-dependent plasticity (Piochon et al.). If parallel fiber EPSPs are elicited in Purkinje cells before activation by the climbing fibers, long-term depression (LTD) will be induced; instead, when they are evoked after climbing fiber activity long-term potentiation (LTP) will occur. As all climbing fibers originate in the inferior olive, this means that the precise timing of activation of olivary neurons is critical. Bazzigaluppi and colleagues did whole-cell recordings of olivary neurons in vivo and showed that the number of wavelets riding on top of their action potentials is related to the amplitude of their subthreshold oscillations as well as the level of electronic coupling between them. The pattern of simple spikes and complex spikes that are generated in the Purkinje cells following various forms of plasticity ultimately converge onto a smaller set of neurons in the cerebellar and vestibular nuclei. Importantly, here these patterns can evoke rebound firing and trigger movements, especially when the timing with respect to the activity of mossy fiber and/or climbing fiber collaterals is optimal (Witter et al.).

## (C) Network patterns

As discussed above, the olivo-cerebellar modules form a unique control system and their specific wiring allows fine temporal control and rhythmicity. Oscillatory and synchronous activities are generated, sustained, and modulated throughout the network, in order to create the appropriate spatiotemporal code necessary to drive behavior.

In his review Rodolfo Llinas focuses on rhythmicity in the olive (Llinas) and he underlies that it is indeed the combination of strong and rather stereotyped intrinsic electrical properties with electrical coupling that allows the synchronous activation of clusters of olivary neurons. Feedback inhibition provides the dynamic variance of the membership of such coupled clusters, and the cluster's activity phase can be resetted by an incoming stimulus or by inputs arising from outside the olivo-cerebellar system.

Geborek and colleagues show that olivary excitability is suppressed during different phases of movement and a relay through the cuneate nucleus is a possible gateway (Geborek et al., Geborek et al.). Elaborating even further on the topic of external inputs providing modulation to system's rhythmicity, Libster and Yarom provide a detailed review of neuromodulators acting on DCN, IO, and PCs and they advocate for the importance of cerebellar neuromodulation, which is necessary to produce a wide range of behavioral response appropriate in the context of the general behavioral state of the animal.

Going back to internal source of rhythmic activity in the olivo-cerebellar system, Person and Ramon contribute with a very comprehensive review on PC-DCN convergence and coding. They underline that disruption to such finetuned code, both in terms of timing and rate, can lead to motor dysfunctions.

Finally, rhythmic activity is not only essential at the input (IO) and output (DCN) stages of the system. Courtemanche and colleagues provide an overview on oscillatory activity of the cerebellar cortex. Slow oscillations (4–25 Hz) organize spatial patterns of synchronization and communication with and within the granular layer. Fast oscillations (150–300 Hz) in PCs have a more direct influence on DCN, neighboring modules and motor output, and are found to be more pronounced in pathological scenarios such as Angelman disease.

## (D) Theories of learning and control

Central to all theoretical models of cerebellar learning is the instructive role played by the IO signals carried via CF to PCs. One of the original proposers of such role, Masao Ito, here elaborates about the apparent dichotomy between sensory (feedforward control) and motor (feedback control) errors carried by CFs to PCs, and pinpoints that such an error dichotomy persists throughout vertebrate phylogeny (Ito). Najafi and Medina focus on the nature of such error signals, and argue that the all-or-nothing idea is being separated. They support this position by underlining that CF burst size has been shown to be tightly regulated and informative, but that it can modulate calcium channels on PC dendrites. A graded CF instructive signal activating PCs can thus be effectively encoded via pre- or post-synaptic modulation. The Otis laboratory confirmed that such signal not only is graded, but is also not univocally received by PCs, but also by MLIs via spillover mechanisms (Otis et al.). Schweighofer et al. discuss the implication of electrical coupling strength in the IO on the error signal effectiveness. They argue that intermediate coupling strength is best, because it leads to chaotic resonance and increase information transfer of the error signal.

Beside the recognized role of supervised plasticity at the PF-CF-PC node, the impact of distributed cerebellar plasticity on cerebellar adaptive behaviors remains to be clarified. This problem would be hard to tackle unless distributed plasticity mechanisms are integrated into a fully interconnected sensory-motor control system operating in closed-loop during behavior. This challenge has been taken by the Ros' and D'Angelo's laboratories (Garrido et al.), who elaborated on a robotic controller, embedding a computational model of the whole olivo-cerebellar system. The model was endowed with multiple distributed forms of synaptic plasticity. During a closed-loop load manipulation task, parallel fiber—Purkinje cell LTP and LTD rapidly acquired sensory-motor contingencies under climbing fiber guidance but then plasticity was slowly transferred into the DCN. This two-rate process proved critical to allow the system to dynamically adjust its gain when the load was changed. Distributed plasticity beyond parallel fiber LTD was therefore required to efficiently generate rapid, stable and self-adapting behavioral learning and control.

## (E) Cerebellum as an integrated system for sensorimotor control and cognition

After the fundamental recognition of its involvement in sensorimotor coordination and learning, the olivo-cerebellar system is now also believed to take part in cognition and emotion. D'Angelo and Casali have reviewed a broad spectrum of observations and argue that a similar circuit structure in all olivo-cerebellar sections may cope with the different cerebellar operations using a common underlying computational scheme. It is proposed that the different roles of the cerebellum depend on the specific connectivity of cerebellar modules and that motor, cognitive and emotional functions are (at least partially) segregated in different cerebro-cerebellar loops. In a multi-level conceptual framework, cellular/molecular and network mechanisms would generate computational primitives (timing, learning, and prediction) that could operate in high-level cognitive processing and finally control mental function and dysfunction. It is proposed that the cerebellum operates as a general-purpose co-processor, whose effects depend on the specific brain centers to which individual modules are connected. Abnormal functioning in these loops could eventually take part in the pathogenesis of major brain pathologies including not just ataxia but also dyslexia, autism, schizophrenia, and depression.

The Apps laboratory highlights anatomical and physiological evidence gathered in monkeys, cats, and rats, indicates that survival circuits structures such as the peri-acquaductal gray are connected with cerebellum and olive (Watson et al.). Additionally, the Rondi-Reig laboratory calls for a key role of the cerebellum in spatial navigation (Rochefort et al.). They argue that the cerebellum is a necessary regulator of spatial representation and integrates multisource self-motion information, transforming such reference frame into vestibular signals and distinguishing between self and externally generated vestibular signals.

## Open questions and debates

While much progress has been made during the last decades in trying to elucidate how the olivo-cerebellar network might work, several questions still remain open. Some of the questions raised by the articles contributing to this special issue concern the genetic and molecular mechanisms during development that generate olivo-cerebellar compartmentalization and determine which behavior is encoded in each cerebellar zone. Another fundamental question is how spatio-temporal patterns elaborated in local microcircuits are integrated into meaningful engrams under the coordination of oscillation and resonance phenomena. At the front of synaptic plasticity there are several open issues. Do we know all existing forms of plasticity in the cerebellum? How do these plasticities contribute to cerebellar learning? How important is structural plasticity in adults and how does this interact with synaptic and intrinsic plasticity mechanisms? How are all the different forms of rhythmicity and plasticity affected by neuromodulators? Taken together, it is becoming clear that understanding how the cerebellum works eventually depends on how its activity is integrated into large-scale loops in the whole brain. A major challenge will be therefore to determine the precise anatomy and behavioral correlates of cerebellar-telencephalic connections in different species, as well as the impact of cerebellar temporally patterned activity onto the cerebral cortex. Tackling such important questions will be the challenge that the field will face in the years ahead of us.

### Conflict of interest statement

The authors declare that the research was conducted in the absence of any commercial or financial relationships that could be construed as a potential conflict of interest.

